# The Eudaimonic Functions of Music Listening Scale: An Instrument to Measure Transcendence, Flow and Peak Experience in Music

**DOI:** 10.3389/fpsyg.2020.566296

**Published:** 2020-09-08

**Authors:** Jenny M. Groarke, Michael J. Hogan

**Affiliations:** ^1^Centre for Improving Health-Related Quality of Life, School of Psychology, Queen’s University Belfast, Belfast, United Kingdom; ^2^School of Psychology, National University of Ireland, Galway, Ireland

**Keywords:** functions of music listening, wellbeing, everyday music listening, psychometrics, scale development

## Abstract

There are many adaptive functions of music listening (AFML) that are relevant for understanding wellbeing. Functions relating to mood and emotion regulation dominate general measures of music listening functions. Eudaimonic functions of music listening (FML) have been identified, but no scale has been developed to measure these functions. The current study reports on the development of a new measure to assess eudaimonic music listening functions. Items were developed based on a prior qualitative study and a literature review focused on music listening and wellbeing. The factor structure was investigated by EFA and CFA in a large sample of participants (*N* = 637, 17–66 years, *M* = 22.04, *SD* = 6.23, 326 males). Tests of dimensionality revealed a three factor scale with seven items. The scale and its subscales possess good internal consistency. The Eudaimonic FML scale measures Transcendence, Flow, and Peak Experience. Contrary to expectations eudaimonic FML did not relate to higher wellbeing (higher positive affect, lower negative affect, higher life satisfaction), rather results suggest that these FML are associated with greater emotional experience more generally. This brief scale will have applications in research focused on music listening benefits, music selection preferences, and experimental and developmental effects of music listening on psychological outcomes.

## Introduction

People report listening to music for many reasons and there are many functions of music listening (FML) that may be adaptive for psychological functioning. The majority of existing measures of music listening have focused exclusively on affect regulation functions. However, in our previous qualitative work when we engaged listeners directly, a broader range of adaptive functions were identified. These included the commonly identified affective (e.g., mood improvement), cognitive (e.g., stimulation), and social functions of music (e.g., connection), but also less commonly identified functions, such as, eudaimonic (e.g., transcendence), goal-attainment (e.g., motivation), everyday listening (e,g., background music), sleep aid functions, and music-focused listening (e.g., appreciation) (Groarke and Hogan, 2016). At the same time, in our efforts to develop a general measure of FML based upon this work, the 11 factors of the adaptive functions of music listening (AFML) scale were predominately affect regulation functions (Groarke and Hogan, 2018). The current study follows on from our previous scale development work and fills an important gap in the literature regarding measurement of a wider range of music listening functions. In particular, the current study focuses on eudaimonic FML.

The rationale for a focus on eudaimonic FML is that these functions deserve special attention due to their significance for music listeners. Specifically, younger and older adults have identified eudaimonic FML (e.g., transcendence, meaning) as adaptive for their wellbeing, and using qualitative structural modeling these functions were seen as influential drivers of other adaptive functions of music, such as, affect regulation (Groarke and Hogan, 2016). Furthermore, existing research (described below) has also identified the potential for eudaimonic activities and experiences to emerge as influential predictors of wellbeing outcomes (Schäfer et al., 2014; van Goethem and Sloboda, 2011).

Previous studies have identified a number of effects of music that can be classified as eudaimonic. These include music-induced flow (Lamont, 2011; Groarke and Hogan, 2016), peak experience (Maslow, 1999; Gabrielsson, 2010), and transcendence (Hays and Minichiello, 2005; Schäfer et al., 2014; Groarke and Hogan, 2016).

**FIGURE 1:**
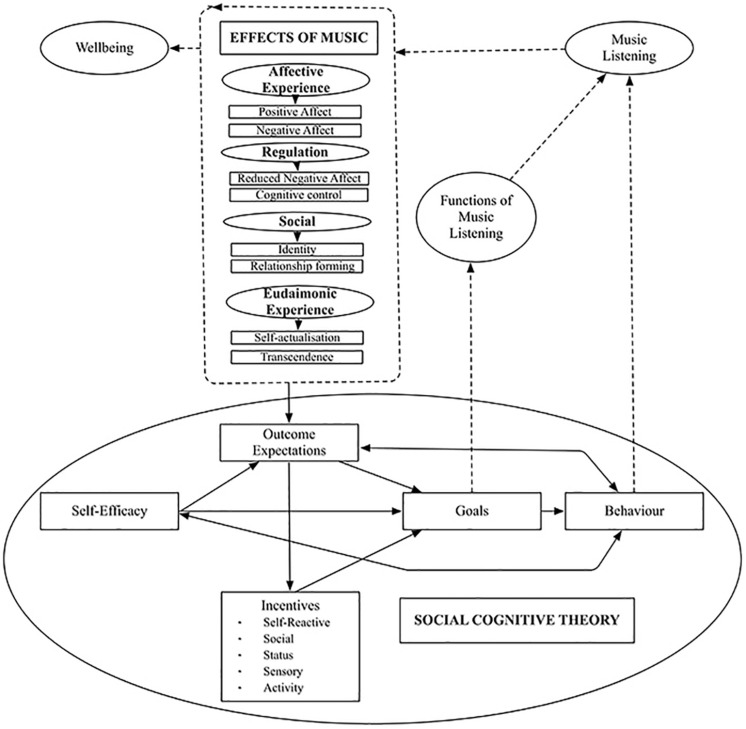
Adaptive effects of music listening identified in the literature linked with functions of music listening via Social Cognitive Theory (Bandura, 1989, 2001).

In line with Bandura’s Social Cognitive Theory (1989, 2001), and consistent with our previous theoretical model focused on the AFML (Groarke and Hogan, 2018), experiencing particular effects of music (e.g., transcendence, flow, peak experience) can create a set of expectations for the listener about the outcomes of listening to music. To the extent that these outcomes are deemed beneficial or provide incentives, outcome expectations become goals that drive music listening behavior (see Figure 1). Within this framework the effects of music are reconceptualized as FML, and FML are related to music listening behavior.

### Peak Experience

Maslow describes peak experiences as a combination of intense positive affectivity, with flow-type states of consciousness, and increased self-realization. Maslow considered peak experiences to be transient episodes of self-actualization. Gabrielsson’s (2010) descriptions of Strong Experiences of Music have much in common with Maslow’s peak experiences. He explains that they are often life-enhancing, giving rise to intense, frequently positive emotional experiences, a change in attitudes and thoughts, spiritual insights and reflections on humanity, momentary loss of self-consciousness, increased hope and self-esteem, and can have therapeutic benefits (Gabrielsson and Lindström, 2001; Gabrielsson and Bradbury, 2011). Schäfer et al. (2014) propose a theoretical model of how intense emotional experiences in music listening facilitate wellbeing. Specifically, the intense musical experience initiates a shift in consciousness where stressors and negative affect are replaced with strong positive feelings. This adjustment in affect is accompanied by cognitive alterations leading to a greater understanding of the self, culminating in a sense of inner harmony and oneness with the world. As a result of the intense musical experience listeners experienced long term changes in their relationships, personal values, meaning, and engagement in life. Given their potential transformative effect, Schäfer et al. (2014) suggest that the intense experience is a key function that is sought out in music listening experiences. Adolescents report using music to generate strong sensations, and seeking intense affective experiences by listening to music (Saarikallio and Erkkilä, 2007), and self-actualization needs are an important function of music listening for both British and American adolescents (Tarrant et al., 2000).

### Flow

The features of these peak experiences during music listening – flow-like states of consciousness, and a reduction of self-consciousness – suggest music may increase engagement in valued activities. Engagement is an important aspect of wellbeing and positive psychological functioning (Csikszentmihalyi, 2002; Seligman, 2012). A diary study by Herbert (2012) found that “effortless engagement” characterized by absorption was commonly reported as part of music listening experiences. Features of absorption included a reduction in the density of thought, alteration in the experience of self, changes in sensory awareness, and greater imaginative involvement in activities. Lamont’s (2011) analysis of descriptions of Strong Experiences of Music listening found that musical episodes were not purely hedonic (increased pleasure and positive affect). Participants also described additional paths to wellbeing in these experiences, particularly through increased engagement. Engagement was described as the occurrence of flow-like states of attention, such as increased focus on the music and reduced attention on surroundings.

### Transcendence

Peak, strong or intense emotional experiences in music listening have also been understood by reference to physiological responses like chills, thrills, and frisson. Such responses have been taken as indicators of transcendent psychophysiological experiences in music (Harrison and Loui, 2014). Salimpoor et al. (2009) found that the chill experience corresponded with subjective pleasure during music listening, and that endogenous dopamine release in the striatum underlies peak emotional responses to music (Salimpoor et al., 2011). Peak experiences may also include experiences of transcendence, which in turn are seen as AFML. For example, in interviews carried out by van Goethem and Sloboda (2011) participants described transcendent effects of music – such as the feeling of being in another world – and these transcendent experiences were seen as important mechanisms in the regulation of affect. In another qualitative study, music listeners identified affect regulation as a consequence of transcendence (Groarke and Hogan, 2016).

### Eudaimonic Functions of Music Listening and Wellbeing

While eudaimonic FML have been identified in the literature, there is currently no scale available to measure eudaimonic functions. Thus, it is unclear how eudaimonic FML relate to wellbeing. That being said, in our qualitative study, participants rated these eudaimonic FML as beneficial for wellbeing enhancement (Groarke and Hogan, 2016). Also, neuroimaging, neurochemical and experimental evidence demonstrates a pattern of positive affective responding in transcendent, peak experiences in music that suggest positive associations between transcendence and subjective wellbeing (SWB) (Blood and Zatorre, 2001; Salimpoor et al., 2009, 2011). The wellbeing benefits of eudaimonic experiences outside of musical contexts have also been widely demonstrated. For instance, transcendence is associated with happiness and meaning (Beaumont, 2009), increased life satisfaction (Gillham et al., 2011), and reduced loneliness (Walton et al., 1991).

### Aims

The current study builds upon our previous scale development work creating a general measure of music listening functions (Groarke and Hogan, 2018) to design a new measure focused exclusively on eudaimonic FML. Drawing upon a specific pool of items emerging from prior qualitative research (Groarke and Hogan, 2016) and a review of the literature, it is hypothesized that three eudaimonic functions will be identified through the process of exploratory and confirmatory factor analysis (CFA): peak experience, flow, and transcendence. Drawing upon a limited body of research on the relationship between eudaimonic FML and wellbeing, positive associations between the three FML factors and SWB measures are hypothesized. This new scale aims to meet the need for a psychometric measure of eudaimonic FML to facilitate further exploration of these functions and their potential adaptive effects, which have been the subject of very little research to date.

## Method

### Initial Scale Development

In earlier work, 164 items were developed based on a comprehensive literature review and focus group sessions with younger and older adults. Items were reviewed by four content experts (three in music psychology, one in psychometrics), and pilot tested with nine lay experts (for full details see: Groarke and Hogan, 2018). The current study is a secondary analysis of 12 items designed to measure three hypothesized eudaimonic FML drawn from the development work for the AFML scale. These 12 items are presented in Supplementary Table 1.

### Design

This study uses a cross-sectional online survey design. The dimensionality of this new measure of eudaimonic FML was identified using exploratory factor analysis (EFA) and CFA. Construct validity was assessed by examining hypothesized positive associations between eudaimonic FML and SWB outcomes (i.e., higher positive affect, lower negative affect, and higher life satisfaction).

### Scale Development

#### Procedure

Potential participants were invited to take part in an online survey of why they listen to music. Recruitment was via university email campaigns, online advertisements, and national media. After reading a participant information sheet outlining the aims of the study and what would be involved in participating, informed consent was provided on the first page of the online survey with the option to withdraw from the study at any time by exiting the survey. If participants did not provide consent, they could not continue to the remainder of the survey. The survey was hosted by Survey Gizmo. Page order of questionnaires was randomized to prevent order effects. The procedure was approved by the National University of Ireland, Galway Research Ethics Committee.

#### Participants

Participants were males or females over 18 years of age. Participants were excluded if they were under 18. 673 participants (452 Females) completed the questionnaire, with 37 participants removed from the dataset for failing to select the correct response to a specific item (i.e., please select the neutral response option), indicating insufficient effort responding. Remaining participants (*N* = 637) were mostly female (68%) university students (98%). Undergraduate psychology students (73%) received research participation credits for participating in the study.

#### Materials

In addition to demographic questions (i.e., age, gender, educational attainment, and employment status), participants completed the following measures:

##### The adaptive functions of music listening scale (Groarke and Hogan, 2018)

Participants rated 164 items measuring 33 hypothesized music listening functions on a five-point Likert scale ranging from 1 (strongly disagree) to 5 (strongly agree). In the current study, responses to 12 items representing three eudaimonic functions of music – peak experience, flow, and transcendence – are analyzed.

##### Music engagement intensity subscale of the music USE questionnaire (Chin and Rickard, 2012)

This eight-item scale provides three measures of music engagement, and allows us to examine relationships between music listening behavior, music listening functions, and wellbeing outcomes. The Index of Music Listening (IML) measures the frequency and duration of music listening. Scores range from 1 to 25, with higher scores indicating more intense music listening. The Index of Music Training (IMT) assesses an individual’s music education and the Index of Music Instrument Playing (IMIP) provides a total score based on the duration and frequency of musical instrument practice. Higher scores indicate more musical training and greater engagement with instrument playing, respectively.

##### Subjective wellbeing

Positive associations between eudaimonic FML and SWB are hypothesized. The components of SWB are both affective and cognitive. More specifically, SWB is defined as high positive affect, low negative affect, and a cognitive evaluation of high satisfaction with one’s life (Diener et al., 1999).

##### Positive and negative affect schedule (PANAS) (Watson et al., 1988)

The PANAS involves self-rating across 20 adjectives: 10 describe positive states and 10 describe negative states. Participants indicate the extent to which they have experienced these affective states in the previous week, using a Likert scale ranging from “very slightly or not at all” (1) to “extremely” (5). Two scores are derived, with higher scores indicating greater positive affect (PA) and negative affect (NA). In the current sample, internal consistency measured by Cronbach’s alpha was high for each subscale: PA (α = 0.88) and NA (α = 0.87).

##### The satisfaction with life scale (SWLS) (Diener et al., 1985)

Using a seven-point scale that ranges from 7 (strongly agree) to 1 (strongly disagree) participants provide ratings of agreement for five life satisfaction statements, such as, “in most ways my life is close to ideal.” Internal consistency of the measure was high in the current sample (α = 0.89).

Descriptive statistics for all of the measures included in this study are presented in Table 1.

**TABLE 1:** Descriptive statistics for variables included in the study, and participant characteristics (*N* = 637).

	Test range	Range	M (SD)
Positive Affect	10–50	10–50	32.23 (7.91)
Negative Affect	10–50	10–48	22.73 (8.19)
Life Satisfaction	5–35	5–35	22.72 (7.18)
Index of Music Listening	1–25	1–25	12.17 (5.75)
Index of Music Training	0–11	2–10	6.75 (1.51)
Index of Musical Instrument Playing		0–575	20.23 (42.37)
Age		17–66	22.19 (6.25)
Gender	Male	32%	
	Female	68%	
Highest level of Education	Would rather not say	0.4%	
	Second level	69.6%	
	Post-secondary	6.4%	
	Undergraduate	15.6%	
	Postgraduate	8%	
Employment status	Would rather not say	0.8%	
	Unable to work	0.2%	
	Homemaker	0.2%	
	Self-employed	0.5%	
	Unemployed	3%	
	Employed	10.2%	
	Student	85.2%	

#### Analysis

To determine the factor structure and reliability of the EFML scale the total sample (*N* = 637) was randomly split in two: EFA was performed on one half of the data (*N* = 298), and CFA was performed on the other half (*N* = 339).

##### EFA

Principal Axis Factoring (PAF) was conducted in SPSS version 23. As factors were hypothesized to be correlated oblique rotation using Direct Oblimin with Kaiser Normalization was used. Following guidelines, items were retained if they had loadings in excess of 0.40, item communalities over 0.40, and no cross-loadings above 0.32 (Worthington and Whittaker, 2006). Factors were examined for the presence of redundant items, with inter-item correlations within factors constrained to be between 0.30 and 0.90. Item-total correlations were required to be above 0.30 to allow for the computation of factor scores without applying item weights (Field, 2009). Factor retention decisions were made on the basis of application of the Kaiser criterion (Eigenvalues greater than 1) (Kaiser, 1960), Horn’s (1965), visual inspection of the Scree plot (Cattell, 1966), maximizing the proportion of variance explained (Beavers et al., 2013), and conceptual considerations.

Confirmatory factor analysis was conducted using Structural Equation Modeling (SEM) in Amos version 23 (Arbuckle, 2014). Measurement models were deemed to provide a good fit to the data on the basis of the following criteria: (a) a non-significant chi square test; (b) the normed chi-square (Q), which is the chi square index divided by the degrees of freedom, is less than 5 (Schumacker and Lomax, 2004); (c) Comparative fit index (CFI) values greater than 0.90 and 0.95, which reflect acceptable and excellent fit to the data, respectively (Kenny and McCoach, 2003); (d) Standardized root mean squared residual (SRMR) and (e) Root mean square error of approximation (RMSEA) values between 0.05 and 0.09 indicating adequate model fit and values below 0.05 indicating excellentfit (Hu and Bentler, 1999).

Scale score reliability was assessed by Cronbach’s (1951) with values of at least 0.70 indicating acceptable internal consistency (Nunnally, 1978).

Construct validity tests were conducted using the entire sample (*N* = 637). As no gold standard measure of eudaimonic FML exists, criterion-related validity could not be assessed. Instead, construct validity was assessed through convergent validity, or how well the scale and subscales converge with measures of related constructs (Furr and Bacharach, 2008). Adopting the view that eudaimonic FML are adaptive, Pearson’s correlations were used to examine associations between factors of the EFML scale and measures of SWB (i.e., higher positive affect, lower negative affect and higher satisfaction with life). The value of the effect size of Pearson’s correlation coefficients varies between −1 and +1, with values of 0.10 indicating a low effect size, 0.30 indicating a medium effect, and 0.50 indicating a large effect (Cohen, 1988).

## Results

### Dimensionality

#### Exploratory Factory Analysis

The Kaiser-Meyer-Olkin measure (KMO = 0.90), and a significant Bartlett’s test of sphericity (*X*^2^_(66)_ = 3229.65, *p* < 0.001) indicated suitability of the dataset for factor analysis. Applying PAF to the 12 item dataset (*N* = 298), two factors were extracted accounting for 45.96% of the variance. Following the iterative removal of four items not meeting retention criteria, a two factor solution accounting for 51.81% of the variance was identified using PAF. Using syntax provided by O’Connor (2000), parallel analysis of the eight item dataset suggested a three factor solution be retained (i.e., the first three eigenvalues for the real data [2.92, 0.93, 0.38] exceeded the first three eigenvalues for the random data [0.36, 0.22, 0.15]). Forcing a three factor solution on this eight item dataset, accounted for 58.11% of the variance. Following the removal of one sub-quality item, and forcing a three factor solution on this seven item dataset accounted for 60.26% of the variance (see Table 2). One item intended to measure Peak Experiences “*Listening to music I can feel a connection with something larger than myself*,” loaded on the Transcendence factor. It was deemed sufficiently conceptually similar to the other items measuring Transcendence to retain the item.

**TABLE 2:** Results of EFA and CFA, psychometric properties of the final seven items and three factors of the EFML scale and factor score intercorrelations in the EFA and CFA samples separately.

	EFA sample (*N* = 298)				CFA sample (*N* = 339)			
		
	Factor 1	Factor 2	Factor 3		Factor 1	Factor 2	Factor 3	
			
Items	Factor loadings				β			*r*^2^
I have had life changing experiences listening to music	0.92				0.88			0.77
I have NOT had a life changing experience as a result of listening to music	0.81				0.85			0.72
I can lose track of time when listening to music		0.74				0.92		0.85
I do NOT lose track of time when I’m listening to music		0.76				0.72		0.52
Listening to music opens up another world of experience			−0.86				0.78	0.61
Listening to music I can feel a connection with something larger than myself			−0.61				0.70	0.49
When listening to music, I feel I can transcend everyday experience			−0.48				0.70	0.49

	**Peak**	**Flow**	**Transcendence**	**Total scale**	**Peak**	**Flow**	**Transcendence**	**Total scale**

Mean (SD)	3.24 (1.09)	3.88 (0.88)	3.84 (0.74)	3.68 (0.69)	3.27 (1.11)	3.98 (0.87)	3.86 (0.79)	3.73 (0.74)
Cronbach’s alpha	0.75	0.56^r^	0.75^r^	0.80	0.75	0.66^r^	0.77^r^	0.85
Variance explained	41.67%	13.23%	5.36%	60.26%				
Eigenvalue	2.92	0.93	0.38					

**Intercorrelations**	**Factor 1**	**Factor 2**	**Factor 3**		**Factor 1**	**Factor 2**	**Factor 3**	

Factor 2	0.216***				0.396***			
Factor 3	0.586***	0.586***			0.630***	0.450***		

Factor 1 = Peak Experience; Factor 2 = Flow; Factor 3 = Transcendence; SD = Standard Deviation; *r* = Pearson’s *r* (two item scales); β = regression coefficient (i.e., factor loading); *r*^2^ = % of variance in the latent variable explained by the item. *p < 0.05, **p < 0.01, ***p < 0.001.

#### Confirmatory Factor Analysis

The three-factor solution identified using EFA was cross-validated using CFA with the other half of the split sample (*N* = 339). The model specified was the seven scale items loading onto their respective factors. This initial measurement model was an excellent fit of the data: *X*^2^_(11)_ = 20.71, *p* = 0.036, *Q* = 1.88, CFI = 0.99, SRMR = 0.026, RMSEA = 0.051 (90% CI, 0.013–0.085), and modification indices did not identify any misspecification in the model. The final seven items of the EFML scale and their beta weights (β), and the proportion of variance in the latent construct explained by items (*r*^2^) in the CFA are reported in Table 2. Finally, CFA using the full sample (*N* = 637) also indicated a very good fit of the measurement model to the data: *X*^2^_(11)_ = 50.33, *p* = 0.000, *Q* = 4.57, CFI = 0.98, SRMR = 0.040, RMSEA = 0.075 (90% CI, 0.055–0.097).

Intercorrelations between the three factors suggest they measure distinct yet interrelated constructs (see Table 2). Factor 1, Peak Experiences, focuses on life changing experiences in music. Factor 2, Flow, taps into losing track of time while listening to music; and Factor 3, Transcendence, measures expansive music listening experiences, such as, entering another world and connecting with something larger than the self.

### Reliability

Results support the reliability of the EFML scale in both halves of the split sample. Inter-item correlations are reported for factors 1 and 2, as it is not appropriate to calculate alpha for two item scales (Streiner, 2003). Cronbach’s alpha coefficients and correlations for each subscale were high suggesting adequate internal consistency of the measure and its subscales (see Table 2).

### Construct Validity

In addition to examining bivariate correlations between EFML factors and SWB measures, a new variable was computed for each factor by multiplying the factor score by scores on the IML. This allows us to analyze relationships between FML and wellbeing outcomes while also considering the intensity of music listening behavior. That is, any differential effect of the music listening function on well-being derives from levels of engagement in music listening.

Scores on the IML were significantly positively associated with total scores on the EFML scale (*r* = 0.342, *p* < 0.001), Peak Experience (*r* = 0.257, *p* < 0.001), Flow (*r* = 0.219, *p* < 0.001), and Transcendence (*r* = 0.331, *p* < 0.001). This pattern of association between FML and music listening behavior is in line with Bandura’s Social Cognitive Theory (SCT) applied to music listening whereby higher expectations of experiencing Peak Experience, Flow, and Transcendence guide FML and predict higher intensity of music listening behavior (see Figure 1). Also consistent with Bandura’s SCT, is the finding that scores on the IML were not significantly associated with SWB [PA (*r* = 0.065, *p* > 0.05), NA (*r* = 0.074, *p* > 0.05), or SWL (*r* = −0.031, *p* > 0.05)], indicating that intensity of music listening behavior alone is not associated with adaptive outcomes.

It was predicted that eudaimonic FML would relate positively to wellbeing outcomes. With the exception of weak correlations with peak experiences (*r* = −0.104, *p* < 0.01) and total scale scores (*r* = −0.079, *p* < 0.05), there were no significant relationships between EFML factor scores and life satisfaction. As shown in Table 3, peak experiences, transcendence and total scale scores were positively associated with both PA and NA. Flow was associated with higher NA only (*r* = 0.093, *p* < 0.05). It is noteworthy that the positive relationship between peak experiences and PA is dependent on intensity of music listening behavior (*r* = 0.088, *p* < 0.05), and the negative associations with life satisfaction are no longer apparent when intensity of music listening is taken into account (*r* = −0.069, *p* > 0.05). However, it should be noted that all effect sizes were low (*r* < 0.20).

**TABLE 3:** Pearson’s correlations between subjective wellbeing (SWB) measures and factor scores, and between SWB measures and factor scores × intensity of music listening behavior in the full sample (*N* = 637).

	PA	NA	SWL
			
	*r*	*r*	*R*
Peak Experience	0.074	0.187***	−0.104**
Peak Experience × IML	0.088*	0.131**	–0.069
Flow	–0.051	0.093*	–0.050
Flow × IML	0.034	0.094*	–0.052
Transcendence	0.133**	0.113**	–0.041
Transcendence × IML	0.100*	0.109**	–0.044
EFML scale total	0.079*	0.165***	−0.079*
EFML scale total × IML	0.081*	0.116***	–0.055

r = Pearson’s correlation coefficient; PA = positive affect, NA = negative affect, SWL = satisfaction with life, IML = index of music listening, *p < 0.05, **p < 0.01, ***p < 0.001.

## Discussion

The Eudaimonic FML scale is a seven item measure composed of three factors: Peak Experience, Flow, and Transcendence. The scale and its subscales possess good internal consistency.

The scale development process followed the steps outlined by DeVellis (2012) thereby ensuring the creation of a high-quality psychometric measure. Following best practice, the scale structure identified using EFA was cross-validated using SEM with data from an independent split sample of participants. This measurement model provided an excellent fit of the data, and was initially grounded in qualitative enquiry, thus increasing the validity of the constructs identified (Padgett, 1998; Rowan and Wulff, 2007). The initial model was refined through literature review and expert review. The criteria applied for the extraction and identification of factors in EFA were well-established and conservative (Costello and Osborne, 2005; Worthington and Whittaker, 2006; Byrne, 2010).

### Eudaimonic Functions of Music Listening and Wellbeing

It was not expected that Peak Experiences and Flow FML would be associated with higher NA – however, in our previous work Reminiscence and Awe and Appreciation functions were also positively associated with NA, and Identity and Strong Emotional Experiences were positively associated with both PA and NA (Groarke and Hogan, 2018). In the current study, Peak Experiences and Transcendence were also related to higher PA and higher NA. One possibility is that the “life changing experiences” captured by the Peak Experience factor are more closely aligned with Gabrielsson’s (2010) strong experiences of music – which can be positive or negative, and are less aligned with peak experiences, which according to Maslow are never negative or unpleasant (Maslow, 1999).

These results suggest that people who endorse eudaimonic FML experience more emotion in general. Perhaps eudaimonic FML relate to wellbeing more broadly, that is, through affective experience rather than affect regulation. In keeping with this view, Labouvie-Vief (2005) describes two pathways to self and emotional development, one involves affect “optimization” (i.e., increasing PA and decreasing NA) and the other involves affect “complexity,” which involves co-ordinating feelings over time and synchronizing with the feelings of others, meaning sometimes positive affect is suppressed and negative affect is maintained – with dynamic integration and trade-offs between the two approaches (Labouvie-Vief and Medler, 2002). FML associated with both PA and NA may be instrumental in shaping integration of optimization and complexity across the lifespan, consistent with adaptive forms of wellbeing characterized by integrated complexity in Labouvie-Vief’s model. This may prove an important avenue for future developmental research focused on the role of music listening in shaping lifespan emotional and personality development.

Bandura’s (1989,^2001^) provided the theoretical underpinning for this scale development project. Applied to music listening this framework conceptualizes effects of music (e.g., transcendence, flow) as functions that drive music listening behavior. In the current study, positive correlations between FML and the IML (i.e., listening duration and frequency) are consistent with this theoretical orientation. Our results also provide some support for the idea that music listening in and of itself (i.e., not linked to any specific function), as measured by the IML is not related to SWB. Based on our previous research, and some of the results in the current study, the role of specific music listening functions are important for understanding relationships with wellbeing outcomes, although further research is needed to investigate these effects. Whether music listening must have a function in order to provide benefit is an important question for future research.

### Future Directions for Research

Eudaimonic experiences have been the subject of very little empirical investigation in music research. Perhaps this is because of the challenge of stimulating these intense, transcendent, and meaningful experiences in laboratory settings, and the lack of assessment tools for measuring these effects of music listening in everyday contexts. This new scale is the first to offer quantitative measurement of a number of eudaimonic FML identified in the wider music psychology and general psychology literature (Maslow, 1999; Gabrielsson, 2010; Lamont, 2011; Schäfer et al., 2014; Groarke and Hogan, 2016). This scale has a number of potential applications. Previous research has found relationships between personality traits, flow (Ullén et al., 2012), and peak experience (Panzarella, 1980; Masluk, 1999). Much research has focused on personality differences in FML (e.g., Chamorro-Premuzic and Furnham, 2007; Vella and Mills, 2017). Future research could also examine how endorsing eudaimonic functions relates to personality traits (e.g., openness) or temperament (e.g., trait anxiety). It could also be used to examine the prevalence of these FML in different populations (e.g., older versus younger adults).

This new scale could be used alongside general measures of music listening functions to profile the prevalence of eudaimonic FML in relation to other perhaps more common FML, such as, affect regulation. This scale could be utilized in more complex designs mapping how different FML relate to one another in driving music listening experiences, or how eudaimonic FML relates to music selection or other context-related music listening variables (see Greb et al., 2018). Used in conjunction with other measures of music listening behavior researchers could examine prevalence-outcome relations, for example, does endorsement of discrete music listening functions coupled with time spent listening to music predict more peak experience, flow or transcendence in context? Finally, the scale could be used as a way to identify people for experimental groupings (i.e., high versus low scores on these functions) before examining effects of music listening interventions on the experience of transcendence, flow, or peak experiences.

### Limitations

The factors representing Peak Experiences and Flow are made up of two indicators each. Although these items possessed good psychometric qualities, in order for factors to be sufficiently identified three to five indicators are recommended (Costello and Osborne, 2005). Therefore, it is not recommended that researchers use these subscales as unidimensional measures. That being said, it is not uncommon to find two-item measures of constructs in larger surveys because of resource or time constraints, or as in the current study, due to considerations of psychometric quality (Eisinga et al., 2013). In the context of large questionnaire batteries the brief nature of this scale allows for rapid data collection on eudaimonic FML. Further, the way these two factors converged as one positively phrased item and its negatively phrased alternate item may be somewhat simplistic. Including negatively worded items is recommended to reduce bias (DeVellis, 2012), yet, some argue they do not and should therefore be avoided (van Sonderen et al., 2013).

The scale was developed to measure eudaimonic music listening functions in adults. Scale items were developed in consultation with younger adults (aged 18–30 years) and older adults (aged 60–85 years) (Groarke and Hogan, 2016). However, in the current study, participants were mostly female and mostly university students which may limit the generalisability of the findings to more diverse groups. It is notable that eudaimonic functions of music were found to be more common among older adults (Groarke and Hogan, 2016). It is possible that more positive relations with SWB might emerge with older age groups. Future research is needed to determine the validity of the scale in other age groups, and should also examine differential effects of eudaimonic FML on younger and older adult wellbeing. Gender differences in affective responses generally, and in response to music specifically, are evident but certainly not clear-cut with mixed findings across many studies (e.g., Knight and Rickard, 2001; Fukui and Yamashita, 2003). However, some studies have found that in everyday life females report using music for affect regulation functions more frequently than males (North et al., 2000; Saarikallio, 2008). This may also be true of eudaimonic FML and is worthy of further investigation.

The current study examined relations between eudaimonic FML and SWB outcomes (i.e., positive affect, negative affect, and life satisfaction). Future studies are needed to examine convergence between the EFML scale and wellbeing outcomes that are more closely related to eudaimonia, such as, purpose in life, personal growth, self-acceptance (Ryff, 1989), and identity, meaning, and accomplishment (Seligman, 2012). In a similar vein, these relationships were examined cross-sectionally, and longitudinal studies will be required to establish causality. At the same time, according to Bandura’s SCT which provided the conceptual grounding for scale development, the relationship between music listening functions, behavior, and outcomes is cyclical (see Figure 1). The Eudaimonic FML scale may be a useful addition to longitudinal modeling studies testing these relationships in everyday contexts, perhaps using experience sampling methods and mobile technology.

## Conclusion

The Eudaimonic FML scale offers a robust measure of peak experience, flow and transcendence in music listening. These constructs have been the subject of much important qualitative research in music psychology (e.g., Gabrielsson, 2010; Lamont, 2011; Herbert, 2012; Schäfer et al., 2014; Groarke and Hogan, 2016), and this scale offers, for the first time, a quantitative measure of these FML. This seven-item scale possesses good internal consistency and psychometric characteristics. Further investigations of the scale’s validity are required. Initial tests suggest eudaimonic functions of music are associated with greater affective experience. There is a gap in our knowledge regarding the prevalence and benefits of eudaimonic FML in everyday music listening. We hope this new measure will be useful for researchers working in this area.

## Data Availability Statement

The datasets presented in this article are not readily available because data availability is not covered by the ethics approval. Requests to access the datasets should be directed to JG, j.groarke@qub.ac.uk.

## Ethics Statement

The studies involving human participants were reviewed and approved by National University of Ireland Galway Research Ethics Committee. The patients/participants provided their written informed consent to participate in this study.

## Author Contributions

JG devised the project and collected and analyzed the data. Both authors wrote the manuscript and designed the study.

## Conflict of Interest

The authors declare that the research was conducted in the absence of any commercial or financial relationships that could be construed as a potential conflict of interest.
