# Supplementary material for: The Eudaimonic Functions of Music Listening Scale: An Instrument to Measure Transcendence, Flow and Peak Experience in Music
Source: Front Psychol. 2020 Sep 8;11:566296. doi: 10.3389/fpsyg.2020.566296 (PMC7506155; doi:10.3389/fpsyg.2020.566296)
Supplement: Supplementary file 1 [file Table_1.DOCX]

Supplementary Material

| **Table 1.** Full set of 12 scale development items representing 3 hypothesised eudaimonic functions of music listening. |
| --- |
| **1. Peak Experience** |
| I have had life changing experiences listening to music* |
| I have NOT had a life changing experience as a result of listening to music (RS)* |
| I have completely lost my self in music listening |
| Listening to music I can feel a connection with something larger than myself |
| **2. Flow** |
| I can lose track of time when listening to music* |
| I have had moments of total absorption in music listening |
| I do NOT lose track of time when I'm listening to music (RS)* |
| When listening to music I feel a sense of increased awareness |
| **3. Transcendence** |
| Listening to music opens up another world of experience* |
| Listening to music does NOT take me to another world (RS) |
| Listening to music I can feel a connection with something larger than myself* |
| When listening to music I feel I can transcend everyday experience* |
| *Note;* * = included in the final scale; RS = reverse scored item |
